# Сytotoxic effect of CAR-T cells against modified MCF-7 breast cancer cell line

**DOI:** 10.22099/mbrc.2023.47125.1820

**Published:** 2023

**Authors:** Aigul Kh. Valiullina, Ekaterina A. Zmievskaya, Irina A. Ganeeva, Margarita N. Zhuravleva, Ekaterina E. Garanina, Albert A. Rizvanov, Alexey V. Petukhov, Emil R. Bulatov

**Affiliations:** 1 Institute of Fundamental Medicine and Biology, Kazan Federal University, 420008 Kazan, Russia; 2 Institute of Hematology, Almazov National Medical Research Center, 197341 Saint Petersburg, Russia; 3 Shemyakin-Ovchinnikov Institute of Bioorganic Chemistry, Russian Academy of Sciences, 117997 Moscow, Russia

**Keywords:** Breast cancer, MCF-7, CAR-T, Immunotherapy, Solid tumor

## Abstract

The most often diagnosed and fatal malignancy in women is breast cancer. The International Agency for Research on Cancer (IARC) estimates that there are 2.26 million new cases of cancer in 2020. Adoptive cell therapy using T cells with chimeric antigen receptor shows potential for the treatment of solid tumors, such as breast cancer. In this work the effectiveness of CAR-T cells against monolayer and three-dimensional bioprinted tumor-like structures made of modified MCF-7 breast cancer cells was assessed. The cytokine profile of supernatants after co-cultivation of MCF-7 tumor cell models with CAR-T cells was also measured to reveal the inflammatory background associated with this interaction.

## INTRODUCTION

Most breast cancers are adenocarcinomas, classified based on invasiveness, morphology, and genetic panel analysis, which influence the response to treatment and disease progression [1]. Standard breast cancer treatment is successful in early stages, but it becomes ineffective as the disease progresses. Most early localized malignancies can be treated with surgery alone, even without systemic treatment [2]. Adoptive cell therapy is a highly promising solution to this issue that could be used to treat advanced breast cancers [3]. In adoptive T-cell treatment, tumor-infiltrating lymphocytes or T-cells that have been genetically altered to express novel T-cell receptors or chimeric antigen receptors are transplanted into the patient either autologously or allogeneically. A long-term response and, in some cases, a complete cure, allow us to hope for a positive effect when treating cancer patients. However, despite significant potential in increasing the clinical effectiveness of solid tumor patients, CAR-T cell treatments face several major obstacles [4].

CAR-T therapy is the subject of numerous clinical trials aimed at treating breast cancer. In order to treat relapsed and/or chemotherapy-resistant triple-negative breast cancer (TNBC), the NCT02580747 study seeks to assess the safety of mesothelin-CAR-T cell therapy. To be able to treat patients with metastatic HER2-negative breast cancer, the NCT02792114 study seeks to establish an effective dose of mesothelin-CAR-T cells. Study NCT01837602 investigates the required dose of intratumoral injection of c-Met-CAR-T cells in patients with TNBC. Using autologous MUC1-CAR-T cells in patients with relapsed or resistant TNBC, the phase I/II trial NCT02587689 investigates the efficacy and safety of these cells. In patients with TNBC, the NCT04025216 study is investigating the feasibility, safety, and early efficacy of TnMUC1-CAR-T cells. Finally, treatment with ROR1-CAR-T cells in patients with TNBC is being evaluated for safety in the ongoing clinical trial NCT02706392. Clinical studies utilizing CAR-T cells have become more prevalent, opening up potential avenues for the treatment of solid tumors [5].

## MATERIALS AND METHODS

**Obtaining CAR-T Cells:** The construct encoding the CAR receptor, the packaging plasmid PsPax2 (Addgene plasmid #12260), and the envelope plasmid pMD2.G (Addgene plasmid #12259) were transfected into HEK293T cells using the transfecting agent PEI MAX (Polysciences, USA) to produce lentiviral vector particles. The cells were cultured for 12 hours, and then the media was changed to DMEM with glutamine (Thermo Fisher, USA), 5% fetal bovine serum (FBS), and 5 mmol sodium butyrate (Hyclone, USA), which was then incubated for an additional 28 hours. A sterile membrane filter (Millipore, USA) with a pore width of 0.45 m was used to filter the resultant supernatant, which contained the virus particles. Then, concentration was done using an Amicon Ultra-15, 100 kDa, centrifugal module from Millipore (USA) at 3,000 g for 30 minutes.

According to Kazan Federal University's Local Ethics Committee's approved protocol N27 (28.12.2020), mononuclear cells were extracted from peripheral blood. For that centrifugation was carried out using a Ficoll gradient (PanEco, Russia). The cells were grown in RPMI-1640 media (PanEco, Russia) supplemented with L-glutamine, penicillin/streptomycin, and 10% FBS (Hyclone, USA) at a concentration of 1×10^6^ cells per milliliter. T lymphocytes were chosen and activated using immunomagnetic Dynabeads Human T-activator CD3/CD28 (Invitrogen, USA) particles at a ratio of 2 particles per cell. The RPMI-1640 medium (PanEco, Russia), 10% FBS (Hyclone, USA), 100 U/ml penicillin, 100 g/ml streptomycin, and 300 U/ml recombinant human interleukin 2 (IL-2) (Biotech, Russia) were used to cultivate a subpopulation of T lymphocytes. T cells were exposed to viral transduction 48 hours after activation utilizing circumstances of multiple infection and 50 g/ml protamine sulfate (Sigma-Aldrich, USA). Following transduction, the T cells underwent a further 72 hours of incubation in RPMI-1640 medium (PanEco, Russia). The effectiveness of the lentivirus transduction of T cells was then evaluated using flow cytometry.

**Obtaining Lentiviral Particles:** HEK293T cells were co-transfected with the packaging plasmid PsPax2 (Addgene plasmid #12260), the envelope plasmid pCMV-VSV-G (Addgene plasmid #8454), and a plasmid expressing either the CD19 or pKatushka2S transgene (FP762b, Evrogen, Russia) in order to produce lentiviral particles. In an Optima L-90K ultracentrifuge equipped with Ultra-Clear Tubes 1 × 3 1/2 in. (25 x 89 mm) and a SW28 rotor (Beckman Coulter Inc., USA), the lentiviral particles were collected from the supernatant of transfected cells and concentrated. Following that, tubes containing the concentrated virus particles were stored in the freezer at -80°C.

**Cell Transduction:** In a 12-well plate, the ATCC# HTB-22 MCF-7 breast cancer cell line was plated at a density of 6×10^4^ cells per well. Cells were cultured in a CO_2_ incubator (ESCO, Singapore) for 16-20 hours. Then, 350 ml of the cell culture media were combined with 150 ml of concentrated lentivirus. The media was changed for fresh growth medium after 16 hours of incubation. After 48 hours, transduction effectiveness was evaluated using a CytoFLEX S flow cytometer (Beckman Coulter, USA) to validate the generation of MCF-7(Kat+) cell lines. After that, the lentivirus that encodes the CD19 antigen was introduced to some of the transformed cells. The generation of MCF-7(Kat+CD19+) cell lines was once again confirmed following 48 hours of transduction efficiency testing utilizing a CytoFLEX S flow cytometer. Lentiviral transduction was also used to generate MCF-7(CD19+) cells that were used for xCELLigence real-time biosensor cell analysis.

**Evaluation of CAR-T Cell Cytotoxicity Against Modified MCF-7 Tumor Cells Using xCELLigence Biosensor Analyzer:** The xCELLigence biosensor cell analyzer (ACEA Biosciences, USA) was used in real-time mode to monitor the proliferative activity of MCF-7 and MCF-7(CD19+) cells. To track proliferation, tumor cells were placed on an E-plate 16 (ACEA Biosciences, USA) with 5×10^3 ^cells per well of RPMI-1640 culture media. CAR-T or T cells (control) were then added to the relevant wells, and the incubation process proceeded until the MCF-7(CD19+) cells finally died, which happened at 98 hours for both MCF-7 and MCF-7(CD19+) cells. The cell index was automatically registered every 15 minutes.

**Evaluation of CAR-T Cell Efficacy Against Modified MCF-7 Tumor Cells Using Confocal Scanning Microscopy:** MCF-7(Kat+) and MCF-7(Kat+CD19+) tumor cells were plated at 5×10^4^ cells per well on the culture plate to assess the capacity of CAR-T cells to eradicate them. 1×10^5^ CAR-T cells were added to each well after 12 hours, and the cells were then kept incubating. Confocal scanning microscope LSM 700 (Carl Zeiss, Germany) was then used to dynamically observe the cell cultures. On days 2, 5, and 7 of the cultivation, at least six representative micrographs of the examined cell cultures were taken. To determine the proportion of the area filled by MCF-7(Kat+) and MCF-7(Kat+CD19+) cells, the micrographs were processed using the ImageJ program.

The effectiveness of CAR-T cells against 3D tumor cell cultures was evaluated. 3D in vitro tumor model consisting of a 5 mm wide and 3 mm high cylinder was used to assess the capacity of CAR-T cells to invade three-dimensional tumor-like structures. The model was created using the Blender program, then Sublime Text 3 was used to modify it for printing in a 96-well plate format. Using MCF-7(Kat+) and MCF-7(Kat+CD19+) cells at 1107 cells per ml, the 3D in vitro tumor model was bioprinted using the Inkredible bioprinter (CELLINK, Sweden) and bioink CELLINK A-RGD in accordance with the manufacturer's instructions. CAR-T cells were added to the culture medium at 1×10^6^ cells per well after 24 hours of incubation. Every day, 50% of the culture media was changed with new medium, and the collected material was used for multiplex cytokine and chemokine analysis. Using confocal scanning microscope LSM 700 (Carl Zeiss, Germany) in 3D mode the daily dynamic observations were carried out to determine the nature of the interaction between CAR-T cells and the tumor-like formations. Using the Las Ez 4.0 tool, fluorescence micrographs were taken and cross-section analyses of 3D cultures of tumor cells stained with hematoxylin and eosin were performed to determine the degree of CAR-T cell penetration.

**Multiplex Analysis of Supernatants for Cytokines** **:** The Bio-Plex Pro Human Cytokine Panel kit, a 17-plex assay (Bio-Rad, USA), was used in accordance with the manufacturer's instructions to perform multiplex analysis of cytokines and chemokines supernatant after the co-incubation of CAR-T and tumor cells.

Data analysis was performed using SPSS v17.0 program, Student's t-test, Mann-Whitney test for two independent samples, and Kruskal-Wallis test were used to compare the groups. Statistics were regarded to be significant when a difference had a p value of 0.05 or below. The statistical significance of variations in CAR-T cell penetration depths into 3D tumor cell structures throughout one culture period was assessed using the one-way analysis of variance.

## RESULTS AND DISCUSSION

**Figure 1:**
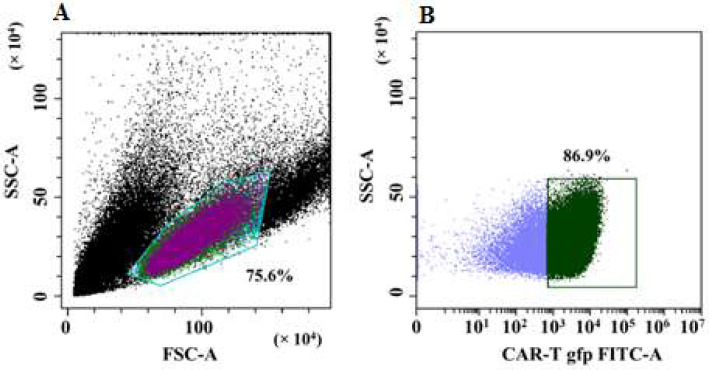
Evaluation of T-lymphocytes transduction efficiency by lentiviral particles encoding CAR. A – dot plot: T cell population on the forward and side scatter plot. B – dot plot: population of GFP+ transduced T-lymphocytes (CAR-T cells)

**Figure 2:**
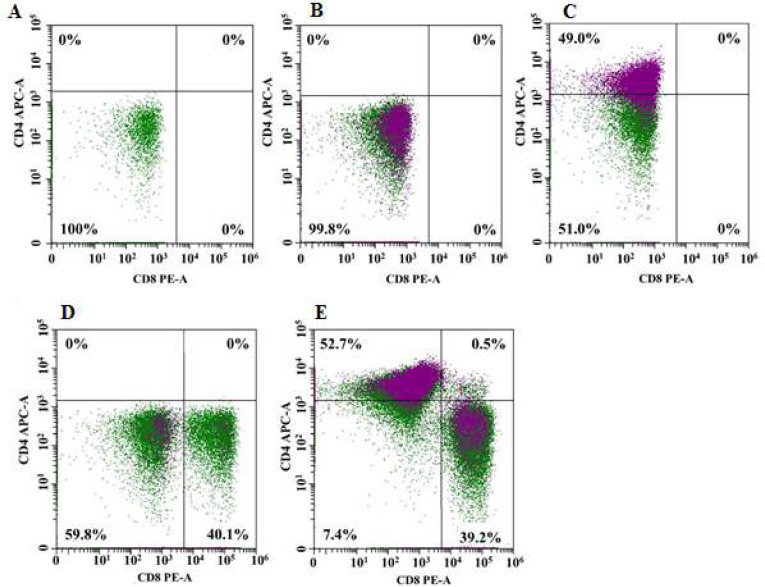
Assessment of the distribution of CD4+ and CD8+ subpopulations in the total population of CAR-T cells by flow cytometry. The figure shows representative distribution plots of unstained GFP+ CAR-T cells (green) and stained with biotinylated protein L and streptavidin-Pacific Blue conjugate (purple). A– unstained control CAR-T cells; B – cells stained with protein L and streptavidin-Pacific Blue conjugate; C– fluorescence control stained with a single labeled anti-CD4 antibody (APC-A); D– fluorescence control stained with a single labeled anti-CD8 antibody (PE-A); E– subpopulation of CD4+ (upper left square) and CD8+ (lower right square) cells in the total population of CAR-T cells

The level of the GFP+ signal was used to measure the effectiveness of transducing T-lymphocytes with a lentivirus expressing CAR, and was found to be equal to 86.9% (Fig. 1 B). Based on the intensity of the associated antibodies fluorescence, the ratio of CD4+ and CD8+ T cell subpopulations was calculated. The percentages of CD4+ and CD8+ CAR-T cell populations in the overall CAR-T cell population were 52.7% and 39.2%, respectively (Fig. 2C-E). The findings point to a significant proportion (up to 86.9%) of T cells in the study population that are chimeric antigen receptor-bearing, as well as the existence of CD4+ and CD8+ CAR-T cells that perform effector roles for the destruction of tumor cells. These results are in line with previously published research, which estimated that 73% of peripheral blood T cells might be transduced by CAR lentivirus [6].

The depletion process must be taken into account when analyzing CD4+ and CD8+ T cells. Although comparable to those of CD8+T cells, the regulatory mechanisms involved in the depletion of CD4+T cells requires further investigations. Although equally significant, CD4+ T cell depletion has received less attention from the scientific community and should be taken into consideration when developing novel immunotherapeutic drugs [7].

According to prior research, the average CD4+ CAR-T cell count was about 50% and the average CD8+ CAR-T cell count was around 27% [8]. Furthermore, it has been shown that CD8+ T cells' ability to specifically lyse target cells is largely a result of the functional expression of granzyme B and perforin, whereas CD4+ T cells play a crucial role in the regulation and maintenance of immune system cells, including CD8+ T cells, through IL-2 secretion. These populations are crucial to CAR-T therapy because prior research has shown that the CD4+ and CD8+ T cell populations contain functionally and transcriptionally distinct subpopulations of cells that vary in their capacity to proliferate and persist in vivo after in vitro expansion and adoptive transfer [9,10]. Additionally, a preclinical model demonstrated that human CD19-CAR-T cells generated from CD8+ or CD4+ T cells were superior to a mixed population of CAR-T cells in their ability to eradicate CD19+ malignancies in immunodeficient mice [11].

**Figure 3:**
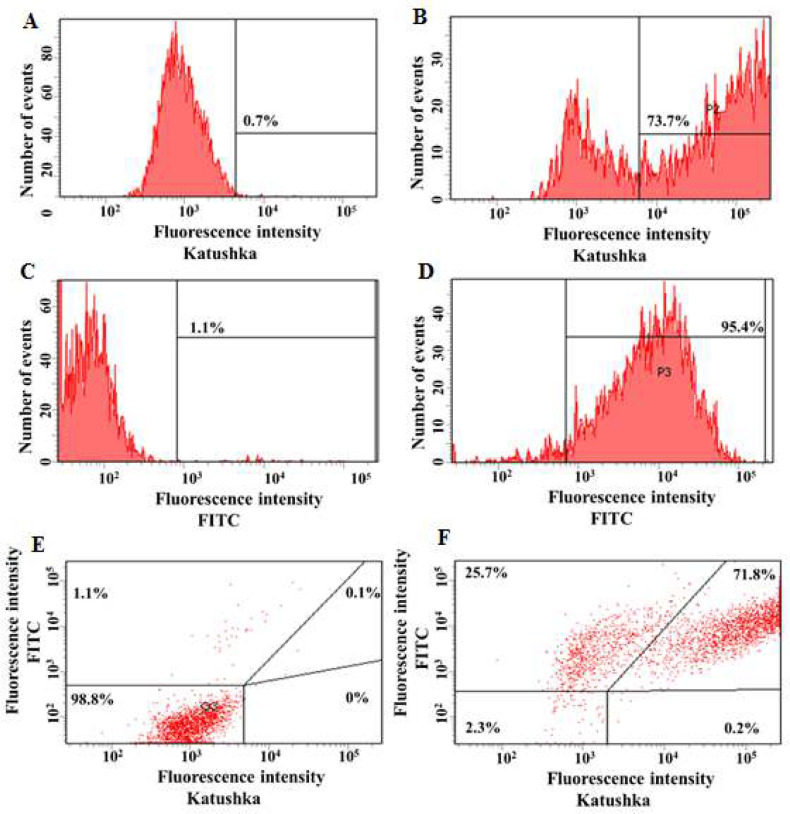
Evaluation of the transduction efficiency of MCF-7 cells with lentiviruses encoding Katushka and/or CD19. Flow cytometry, representative histograms (A-D), and dot plots (E, F) are presented. A – negative control, non-transduced cells; B–MCF-7(Kat+) cells expressing red fluorescent protein Katushka; C–negative control, non-transduced cells; D–MCF-7(CD19+) cells expressing the CD19 antigen; E–negative control, non-transduced cells are shown in the upper right corner; and F–MCF-7(Kat+CD19+) cells simultaneously expressing both red fluorescent protein Katushka and the CD19 antigen are shown in the upper right corner

The ability of MCF-7 breast cancer cells to be transduced by the lentivirus that encodes Katushka red fluorescent protein, was found to be 73.7%, whereas the background fluorescence in the non-transduced control sample was only 0.7% (Fig. 3 A,B). Furthermore, MCF-7(Kat+) cells were transduced with lentiviruses that encode CD19 to produce MCF-7(Kat+CD19+) cells (71.8%), compared to a control sample of non-transduced cells (0.1%) (Fig. 3 E,F).

**Figure 4:**
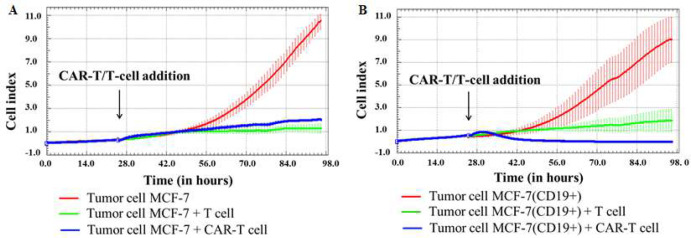
Evaluation of the cytotoxicity of CAR-T cells against MCF-7 and MCF-7(CD19+) tumor cell lines using xCELLigence real-time biosensor cell analysis. Dynamic monitoring of proliferation of MCF-7 and modified MCF-7(CD19+) cells with and without the addition of T/CAR-T cells. Graphs of cell index versus time are presented as mean, the error bars indicate standard deviation (n=3). A – MCF-7 cells; B – MCF-7(CD19+) cells

After the addition of CAR-T cells, real-time biosensor cell analysis utilizing the xCelligence system showed a substantial decrease in the proliferative activity of MCF-7(CD19+) tumor cells, with a significant drop when compared to the control unmodified MCF-7 cells. Interesting to note that the presence of T lymphocytes also caused the death of MCF-7(CD19+) cells comparable to that of CAR-T sample (Fig. 4). These findings suggest the possibility of nonspecific partial cytolysis of CD19+ tumor cells T lymphocytes. This data analysis is compatible with previously obtained results indicating that both anti-CD19 CAR-T and activated T cells displayed comparable specific cytotoxicity against breast, pancreatic, ovarian, prostate, and lung cancer cell lines, ranging from 10% to 90% [12].

**Figure 5:**
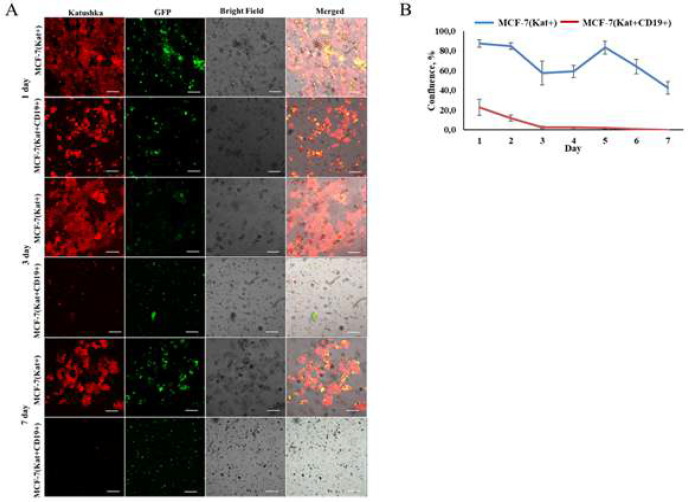
Fluorescence microscopy evaluation of the effectiveness of CAR-T cells against modified tumor cells over a 7-day period. A – Micrographs of MCF-7(Kat+) and MCF-7(Kat+CD19+) tumor cell monolayers treated with CAR-T cells for a period of 7 days. Light microscopy, fluorescence microscopy, and representative micrographs (n = 6) are shown. Scale bar is equal to 100 µm. CAR-T cells – green fluorescence; MCF-7(Kat+) and MCF-7(Kat+CD19+) cells – red fluorescence; B – Plot of comparative confluence of MCF-7(Kat+) (blue line) and MCF-7(Kat+CD19+) (red line) cells after addition of CAR-T cells

The dynamic observation of MCF-7(Kat+CD19+) cells treated with CAR-T cells revealed a considerable, gradual reduction in the confluence of the tumor cell monolayers (Fig. 5). Contrarily, for the whole 7-day incubation period, the addition of CAR-T cells to the control MCF-7(Kat+) cell line did not result in a continuous decrease in the tumor cells confluence. The micrographs were analyzed graphically using ImageJ software.

**Figure 6:**
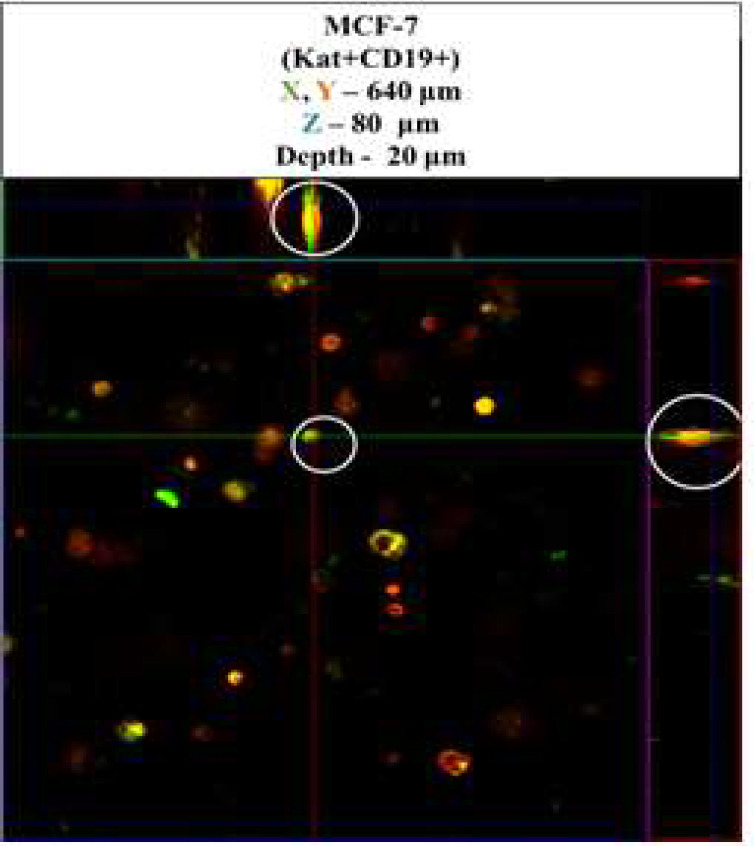
Orthotopic micrograph of 3D structures formed by tumor cells MCF-7 on the 4^th^ day after addition of CAR-T cells. Confocal fluorescence microscopy and representative micrographs are shown (n = 6). Circles indicate examples of single CAR-T cells (green fluorescence) and areas of their direct contact with tumor cells (red fluorescence).

The effectiveness of CAR-T cells against 3D cultures of tumor cells was assessed by using a confocal scanning microscope LSM 700 (Carl Zeiss, Germany) in 3D mode to dynamically observe the tumor-like formations. On the fourth day of co-culturing CAR-T cells deeply penetrated the 3D tumor-like formations, as shown by the confocal microscope analysis results in (Fig. 6). The circles in the figure specify the locations where tumor cells and CAR-T directly interact. Our results show that CAR-T cells were present at different levels of the matrix depth, including right next to tumor cells, and could penetrate the three-dimensional tumor-like structures formed by MCF-7(Kat+) and MCF-7(Kat+CD19+).

A previous study discovered that within 72 hours of addition CAR-T cells could directly interact with tumor cells and significantly infiltrate the Gelatin Methacryloyl (GelMA) hydrogel [13]. In our recent work, we used four cell lines – lung adenocarcinoma (H522), prostate cancer (PC-3M), epidermoid carcinoma (A431), and triple-negative breast carcinoma (MDA-MB-231) – to assess the effectiveness of CAR-T cells against modified CD19+ solid tumor models [14]. 

**Figure 7:**
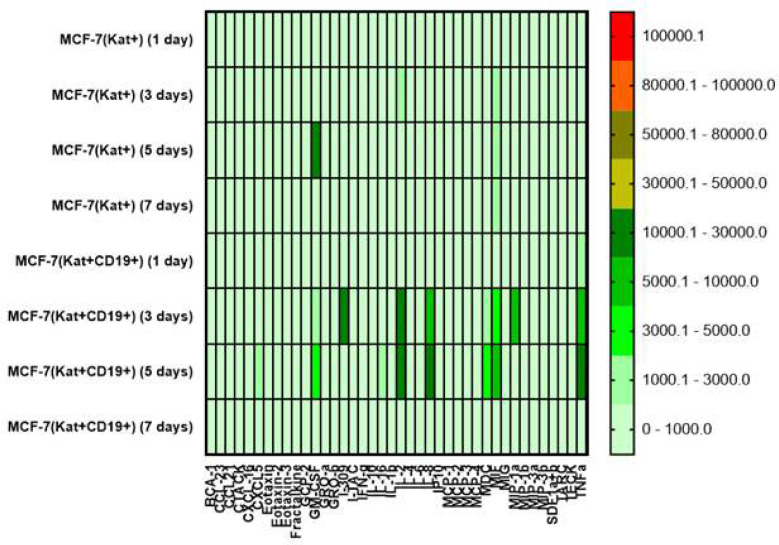
Heat map of cytokines and chemokines multiplex arrays in the culture medium after co-cultivation of CAR-T cells with MCF-7(Kat+) and MCF-7(Kat+CD19+) tumor cells at different time points (days 1, 3, 5, and 7).

According to cytokine analysis the level of IL-8 secretion increased on the 3^rd^ day following the addition of CAR-T cells in co-culture with MCF-7(Kat+CD19+) tumor cells, and then dropped on 7^th^ day. On days 3-5, MIF, TNF, and IL-2 secretion was also increased (Fig. 7).

The results support the previous findings that CAR-T cells can exhibit heterogeneity in cytokine production. The responses ranged from stimulatory (GM-CSF, IL-2, and IL-8) to antitumor effector (Granzyme B, IFN, MIP1, and TNF), regulatory (IL-4, IL-13, and IL-22), as well as inflammatory (IL-6 and IL-17A) [15].

## Conflict of Interest:

The authors declare no conflict of interest.
